# Behavioral and Metabolic Effects of a Calorie-Restricted Cafeteria Diet and Oleuropein Supplementation in Obese Male Rats

**DOI:** 10.3390/nu13124474

**Published:** 2021-12-15

**Authors:** Alex Subias-Gusils, Adam Álvarez-Monell, Noemí Boqué, Antoni Caimari, Josep M. Del Bas, Roger Mariné-Casadó, Montserrat Solanas, Rosa M. Escorihuela

**Affiliations:** 1 Institut de Neurociències, Universitat Autònoma de Barcelona, 08193 Bellaterra, Spain; Alex.Subias@uab.cat (A.S.-G.); Adam.Alvarez@uab.cat (A.Á.-M.); 2 Departament de Psiquiatria i Medicina Legal, Facultat de Medicina, Universitat Autònoma de Barcelona, 08193 Bellaterra, Spain; 3 Department of Cell Biology, Physiology and Immunology, Faculty of Medicine, Universitat Autònoma de Barcelona, 08913 Bellaterra, Spain; 4 Eurecat, Centre Tecnològic de Catalunya, Technological Unit of Nutrition and Health, 43204 Reus, Spain; noemi.boque@eurecat.org (N.B.); antoni.caimari@eurecat.org (A.C.); josep.delbas@eurecat.org (J.M.D.B.); roger.marine@eurecat.org (R.M.-C.); 5 Eurecat, Centre Tecnològic de Catalunya, Biotechnology Area and Technological Unit of Nutrition and Health, 43204 Reus, Spain

**Keywords:** diet-induced obesity, energy restriction, food intake, hedonic response, leptin, polyphenols, sucrose preference, sweet taste

## Abstract

Diet-induced obesity models are widely used to investigate dietary interventions for treating obesity. This study was aimed to test whether a dietary intervention based on a calorie-restricted cafeteria diet (CAF-R) and a polyphenolic compound (Oleuropein, OLE) supplementation modified sucrose intake, preference, and taste reactivity in cafeteria diet (CAF)-induced obese rats. CAF diet consists of high-energy, highly palatable human foods. Male rats fed standard chow (STD) or CAF diet were compared with obese rats fed CAF-R diet, alone or supplemented with an olive tree leaves extract (25 mg/kg*day) containing a 20.1% of OLE (CAF-RO). Biometric, food consumption, and serum parameters were measured. CAF diet increased body weight, food and energy consumption and obesity-associated metabolic parameters. CAF-R and CAF-RO diets significantly attenuated body weight gain and BMI, diminished food and energy intake and improved biochemical parameters such as triacylglycerides and insulin resistance which did not differ between CAF-RO and STD groups. The three cafeteria groups diminished sucrose intake and preference compared to STD group. CAF-RO also diminished the hedonic responses for the high sucrose concentrations compared with the other groups. These results indicate that CAF-R diet may be an efficient strategy to restore obesity-associated alterations, whilst OLE supplementation seems to have an additional beneficial effect on sweet taste function.

## 1. Introduction

The prevalence of overweight and obesity are escalating worldwide, along with Metabolic Syndrome (MetS) and the development of type-2 diabetes and cardiovascular diseases (CVD) [1,2]. Among the alterations associated with obesity, changes in taste function are supported by a robust body of evidence. Psychophysical studies have demonstrated that sensory and hedonic properties of sweet and fat vary with body mass index in humans, in a manner such that obese people experience reduced sweetness and intensified fat sensations, liking both sweet and fat more than the non-obese do [3]. Moreover, sensory taste (including sweetness, saltiness, sourness, umaminess, bitterness, and oleic acid, a fatty stimulus), olfactory function, and eating behavior have been shown to improve after bariatric surgery [4]. In a recent meta-analysis investigating how physiological factors, pathologies and acquired habits influence taste sensitivity, the only factor increasing the sucrose detection threshold was a high body mass index (BMI), while aging and type 2 diabetes mellitus patients were found to exhibit an increased sucrose recognition threshold [5]. However, whether these changes are associated with the metabolic consequences of obesity or are the consequence of the sucrose consumption per se remain unclear [6]. The possibility that the taste system can increase or decrease its sensitivity with diet composition and influence food preference, choice, and overall energy intake, through diet-dependent chemosensory mechanisms of plasticity has been proposed [7].

The hormone leptin has been associated with the transmission of sweet taste and with the mechanisms of satiety and neural reinforcement. In humans and rodents with normal body weight, the behavioral response to sweet solutions of different concentrations changes with diurnal variations of plasma leptin levels, while in obese humans and rodents, those diurnal leptin variations were not found [8]. Leptin selectively suppressed sweet taste via the leptin receptor Ob-Rb in sweet taste cells [9]. In humans, it has been also proved that leptin selectively modulates sweet taste neuronal responses and taste perception through activation of oral leptin receptors in taste receptor cells [6].

The most widely used interventions to counteract the impact of overweight and obesity in humans are based on the administration of hypocaloric diets and lifestyle modifications (moderate exercise programs) often combined with bioactive dietary compound supplementation [10,11].

Konstantinidi and Koutelidakis [12] reviewed the contribution of functional foods such as coffee, green tea, berries, nuts, or olive oil, among others, and their bioactive compounds such as caffeine, catechins, gallic acid, anthocyanins, ascorbic acid or oleuropein, among others, to weight management, obesity prevention, and obesity’s metabolism along with their possible mechanisms. They concluded that functional foods, as part of a balanced diet, can be useful for weight management and can decrease obesity’s metabolic consequences, although scientific evidence remains unclear and controversial.

The fruits and oil of the olive tree (*Olea europaea*) are essential components of the Mediterranean diet. Oleuropein (OLE) is a phenolic constituent of olives consisting of three structural subunits: hydroxytyrosol, elenolic acid, and a glucose molecule. OLE and its related compounds have shown to be beneficial against dyslipidemia and to have antiobesity, antidiabetic, antihypertensive, and hepatoprotective actions [13]. The antioxidant, anti-inflammatory, and immunomodulatory properties of secoiridoids from the olive tree such as OLE have potential applications in many inflammatory and reactive oxygen species (ROS)-mediated diseases [14].

Among the animal models developed to study human obesity, the rodent diet-induced obesity (DIO) models have considerable face validity with human obesity and are widely used. More specifically, the cafeteria diet (CAF) better mimics the hedonic hyperphagia that is observed in obese humans and promotes more severe diabetic symptoms than other high fat diets [15]. CAF diet consisting of exposing the rodents to high-energy, highly palatable human foods like cakes, savory snacks, cheese, and sugared milk, induces a rapid weight gain [16,17,18,19] and metabolic disorders associated with obesity and MetS in humans (hyperleptinemia, hypertension, hypertriglyceridemia, hyperglycemia, and insulin resistance) [20,21].

OLE administration prevented body weight gain, body fat accumulation, hyperinsulinemia, and hyperleptinemia in obese mice fed with CAF diet [22] and attenuated visceral adiposity in high-fat DIO mice [23]. OLE also improved the cardiovascular, hepatic, and metabolic signs in high-carbohydrate high-fat fed rats [24] and enhanced fat-oxidation and optimized cardiac energy metabolism in obese rats [25]. In humans, olive leaf tea decreased serum levels of triacylglycerides and low-density lipoprotein cholesterol in prediabetic individuals between 40 and 70 years of age with a body mass index of 23.0–29.9 kg/m^2^ and changed body weight, waist circumference, and insulin levels after a 12-week intervention consisting of consuming 330 mL of the test beverage (consisting of tea leaves steeped in boiled water) three times daily during mealtimes [26].

The current study was aimed to evaluate whether a dietary intervention based on a previously characterized calorie-restricted cafeteria diet (CAF-R) [27], alone or supplemented with OLE, modified sucrose preference, and sweet taste reactivity in cafeteria diet-induced obese rats. We also analyzed the effects on biometric, food and energy intake, and metabolic parameters including leptin levels.

## 2. Materials and Methods

### 2.1. Animals and General Procedures

Forty male Sprague–Dawley rats (23–25 days old) were used (Harlan Laboratories, Barcelona, Spain). The animals were housed 2 per cage at 22 °C under 12 h/12 h light/dark cycle (lights on at 08:00 a.m.) in standard conditions of temperature (21 ± 1 °C) and humidity (50 ± 10%) for the duration of the experiment with free access to food and water. After one week of habituation to the animal facility, the animals were assigned to two treatment groups with equivalent averages of initial body weight: the control group, fed with standard chow ad libitum (STD, *n* = 10), and the CAF group, fed with cafeteria diet ad libitum (CAF, *n* = 30) to induce obesity. After 8 weeks, all animals were housed individually. The STD group was maintained on an ad libitum chow diet, whereas CAF-fed animals were subdivided into three subgroups depending on the dietary treatment received: the remaining cafeteria diet ad libitum group (CAF, *n* = 10); the CAF-R group (*n* = 10), fed with calorie-restricted cafeteria diet; and the CAF-RO group (*n* = 10), fed with CAF-R diet supplemented with olive tree leaves extract (25 mg/kg*day). Once a week, cages were changed and after the cleaning, body weight and food consumption were recorded. Nose:anal length (NAL) was measured once a week from the end of the obesity induction period (week 9) to the end of the experiment.

Two blood samples were collected, one immediately before the dietary treatments started (week 9) and the second one immediately before the behavioral tests (week 19). Serum was obtained by centrifugation at 2000× *g* for 15 min at 4 °C and stored at −80 °C until further analyses. After 22 weeks of dietary treatments, the animals were sacrificed by decapitation after 8 h of fasting.

All animals received human care under an institutionally approved experimental animal protocol, following the legislation applicable in Spain. The experimental protocol was approved by the Generalitat de Catalunya (DAAM 9978), following the ‘Principles of laboratory animal care’, and was carried out in accordance with the EU Directive 2010/63/EU for animal experiments.

### 2.2. Diets

The animals were fed with the corresponding diets daily for 22 weeks, from weaning until the end of the study. Food consumption was calculated as the difference between the weight of each dietary component provided and the amount unconsumed after 24 h.

During the obesity-induction period, the CAF diet daily included (average quantity administered per cage/day) all the following items: bacon (10 g), biscuit with pâté (12 g), biscuit with cheese (13 g), muffins (16 g), carrots (10 g), jellied sugared milk (72 g, 18% sucrose *w*/*w*), and standard chow (25 g). CAF items were placed in a clay pot inside the cage and prepared fresh each day. Mean daily total food and energy administered per cage/day were 158 g/day and 367 kcal/day respectively. The caloric distribution of the CAF diet was 10% protein, 36% fat, and 54% carbohydrates.

During the dietary treatments period, the CAF diet included (average quantity administered per rat/day): bacon (6 g), biscuit with pâté (7 g), biscuit with cheese (7 g), muffins (11 g), carrots (5 g), jellied sugared milk (45 g, 18% *w*/*w*), and standard chow (25 g). Mean daily total food and energy administered per rat/day were 106 g/day and 256 kcal/day, respectively. The caloric distribution of the CAF diet was 11% protein, 34% fat, and 55% carbohydrates. Both CAF-R and CAF-RO diets were based on the same items and had a very similar qualitative composition to the CAF diet, but the amount of each food item administered was readjusted every week with a 30% calorie restriction relative to the energy intake consumed by the CAF group. Averaged over the 14 weeks, CAF-R and CAF-RO diets included (average quantity administered per rat/day): bacon (3.2 g), biscuit with pâté (3.4 g), biscuit with cheese (6.4 g), muffins (3.2 g), carrots (3.8 g), jellied sugared milk (12.1 g, 18% *w*/*w*), and standard chow (13.2 g). Mean daily total food and energy administered per rat/day were 45.3 g/day and 115 kcal/day, respectively. The caloric distribution of this diet was 12% protein, 35% fat, and 53% carbohydrates.

The STD group was fed with standard chow (Teklad Global 14% Protein Rodent Diet 2014, Harlan) ad libitum during the entire experiment. Mean daily total food and energy administered per rat/day were 25 g/day and 72.5 kcal/day. The caloric distribution of the STD diet was 20% protein, 13% fat, and 67% carbohydrates.

### 2.3. Oleuropein Supplementation

A 25 mg/kg daily dose of an *Olea europaea* leaf extract (Benolea^®^, Frutarom Health BU, Switzerland), containing a 20.1% of OLE, was administered to the CAF-RO-fed animals. The extract was diluted with sugared (0.7% *w*/*w*) strawberry gelatin to a concentration of 3 mg/mL gelatin and was prepared and administered daily for the 14 weeks of the dietary treatments to the CAF-RO group. Simultaneously, a placebo strawberry gelatin without OLE was prepared and administered daily for the rest of the groups (STD, CAF and CAF-R) as a control procedure. During the 14 weeks of OLE/placebo sugared gelatin supplementation the animals were fed with their corresponding diets.

### 2.4. Body Mass Index

Body length was measured as the NAL in order to estimate the BMI using the following formula: weight (g)/body length (cm)^2^.

### 2.5. Serum Analyses

An enzymatic colorimetric kit was used to assay serum triacylglycerides (QCA, Barcelona, Spain). Serum insulin and leptin levels were measured using a mouse/rat insulin ELISA kit (Millipore, Barcelona, Spain) and a rat leptin ELISA kit (Millipore), respectively. Insulin resistance was estimated using the homeostatic model assessment (HOMA-IR), following the formula: HOMA-IR = Glucose × Insulin/22.5 [28].

### 2.6. Behavioural Procedures

#### 2.6.1. Two-Bottle Preference Test

To determine the preference for sucrose solutions, a two-bottle preference test was performed from weeks 19 to 21. It consisted of placing two bottles at the opposing sides of the food container cover of the animals’ cage, one containing a sucrose solution and the other one containing tap water. First, the animals were presented with tap water from two bottles to habituate them to the procedure and as a control to measure water intake for 48 h. After the habituation period seven concentrations of sucrose solutions were tested (0.01 M, 0.03 M, 0.06 M, 0.1 M, 0.3 M, 0.6 M, 1 M), presented in ascending order. Sucrose solutions were prepared daily using tap water. Each sucrose solution was presented for 2 days/48 h for a total of 14 days together with a bottle of water. Water and sucrose intakes were recorded daily and bottle positions were switched every 24 h to avoid laterality preference [29]. Sucrose preference ratio was calculated according to the formula: sucrose solution intake (g)/water + sucrose solution intake (g). All animals were fed with their corresponding diets throughout the behavioral test.

#### 2.6.2. Taste Reactivity Test

To evaluate hedonic responses to different sucrose solutions, we performed the taste reactivity test for 4 days at week 22 of the experiment. On the first day of the test, the animal was placed in a cylindrical cage for 5 min without solution to habituate it to the test conditions. The next day the animal was placed in the cylindrical cage and after 2.5 min of habituation to the testing place, 1 mL of tap water was deposited on the floor to habituate it to the presence of a drop of solution. After these acclimation days, the third and the fourth day 1 mL of sucrose solution (0.1 M and 1 M) was deposited on a corner of the transparent floor after an acclimation period of 2.5 min. The orofacial expressions were recorded from below using a video camera (JVC, HD Everio GZ-HD10) during and after voluntary sucrose intake for 2.5 min. Sucrose solutions were prepared daily using tap water. Positive hedonic responses were measured as number of episodes (n), and tongue protrusion (TP), paw licking (PL) and mouthing (M) were measured. Aversive responses were measured as the number of episodes, and included head shaking (HS), forelimb flailing (FF), snout cleaning (SC) and gagging (G). Both types of responses were considered by examination of slow-motion recorded videos [30]. This test was performed similarly as described by Shin et al. 2011 [31].

### 2.7. Statistical Analyses

Statistical analyses were performed using SPSS Statistics 22 (SPSS, Inc., Chicago, IL, USA). The homoscedasticity among groups was measured using Levene’s test. The normal distribution of the values was measured using the Kolmogorov-Smirnov test. A Mann-Whitney U test was performed to evaluate differences between groups on biometric, food intake and serum parameters at the end of the obesity-induction period due to the different sample size of STD and CAF groups. One-way analysis of variance (ANOVA) followed by Tukey post-hoc or Student’s *t* tests were performed to evaluate differences among groups on biometric, food intake and serum parameters at the end of the dietary treatments period. Triacylglycerides and leptin data were log transformed to homogenize variances. The intake data for sucrose solution, total fluid and total sucrose were analyzed by a Repeated Measures ANOVA, with the sucrose solution concentration as the within-subjects factor and the diet as the between-subjects factor. Wilks’ Lambda of Multivariate tests was used to detect significant sucrose solution effects and significant ‘sucrose solution × diet’ interactions. Sucrose preference was analyzed with a Kruskal-Wallis test followed by a Mann-Whitney U test. Data from taste reactivity test was analyzed using Student’s *t*-test for comparisons between groups. Spearman’s rho correlation was performed to determine the relationship between serum leptin levels and sucrose preference and total sucrose consumption. All the results were expressed as mean ± SEM. The level of statistical significance was set at bilateral 5%.

## 3. Results

### 3.1. Effects of Diets on the Biometric, Food Intake, and Serum Parameters

Biometric, average daily intake of food, energy and simple sugars, and serum parameters (Table 1) were measured at the end of the obesity-induction period and after 10 weeks of dietary treatments (weeks 9 and 19, respectively). At week 9 the CAF animals showed a higher increase of body weight and BMI (*p* < 0.01) than the STD animals. The CAF group also showed higher values of daily food and energy intake than the STD group during that period (*p* < 0.001). Only the CAF group consumed simple sugars due to the presence of high-sugar food components in this diet. As for the serum metabolic parameters, the CAF diet induced an increase in circulating levels of triacylglycerides, insulin, insulin resistance indicated by the HOMA-IR (*p* < 0.05), and leptin (*p* < 0.001), compared to the STD group.

At week 19 the results showed that CAF-fed animals had a greater body weight gain than the other three groups (*p* < 0.001). CAF-R and CAF-RO groups showed a significantly lower weight gain compared with the CAF group, whereas no differences were observed with the STD group or between them. Similarly, the CAF group showed a significantly increased BMI compared to the other three groups at this time, while no differences were observed between the STD and CAF-R or CAF-RO groups (*p* < 0.001). In addition, the CAF-fed animals showed the highest values of food and energy intake, the STD the lowest values, and CAF-R and CAF-RO animals intermediate values (*p* < 0.001). Both CAF-R and CAF-RO-fed animals consumed less than half the amount of simple sugars consumed by CAF-fed animals (*p* < 0.001).

Regarding the serum parameters, the CAF and CAF-R groups showed higher levels of circulating triacylglycerides compared with the STD group (*p* < 0.001 and *p* < 0.05, respectively), whereas CAF-RO showed no differences from STD. The CAF group also showed a significant increase in insulin levels (*p* < 0.05), whereas no differences appeared in CAF-R and CAF-RO groups, compared to the STD group. Both CAF and CAF-R diets increased insulin resistance compared to STD diet (*p* < 0.05), whereas the CAF-RO diet did not change the HOMA-IR compared with the STD diet. Finally, the CAF, CAF-R and CAF-RO diets increased leptin levels compared to the STD diet (*p* < 0.001).

### 3.2. Two-Bottle Preference Test

#### 3.2.1. Sucrose Solution Intake and Preference

**Figure 1:**
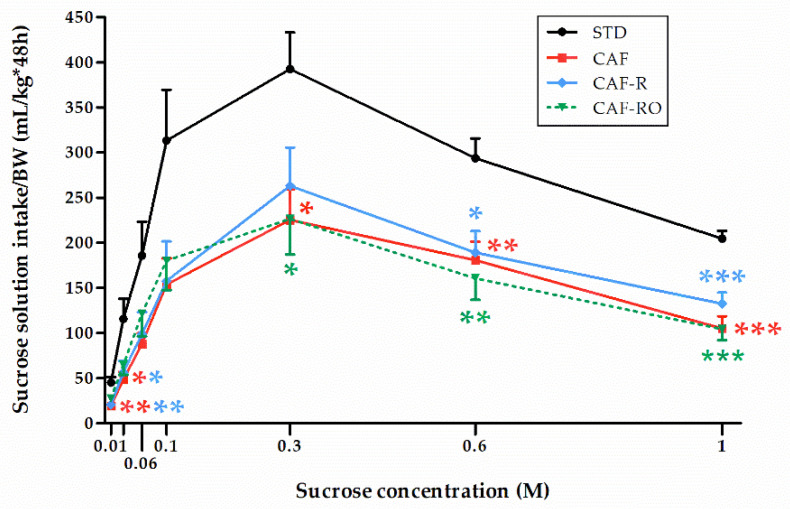
Effects of the dietary treatments on sucrose solution intakes in the two-bottle preference test. Sucrose solution intakes were relativized to the body weight (BW, kg). Data are expressed as the mean ± SEM. * *p* < 0.05, ** *p* < 0.01, *** *p* < 0.001 vs. STD group.

To determine the preference for different sucrose solutions, a two-bottle preference test using several concentrations of sucrose (0.01 M, 0.03 M, 0.06 M, 0.1 M, 0.3 M, 0.6 M, 1 M) was performed (Figure 1). Results revealed a significant effect of concentration on the sucrose solution intake (*p* < 0.001), and that this effect was dependent on the dietary treatment received (‘sucrose concentration × diet’, *p* < 0.05). The sucrose solution intake followed an inverted-U pattern in all experimental groups. They gradually increased the intake over 0.01 M–0.3 M solutions, reaching the maximum of the inverted curve at 0.3 M. From that point on, all groups gradually decreased the intake over the 0.6 M and 1 M solutions with a steep or gentle slope depending on the group. The STD group showed the highest intakes at all sucrose solution concentrations. In fact, the sucrose solution intake of the STD rats almost doubled the intake shown by the cafeteria rats, not showing differences among them at any of the sucrose concentrations.

**Figure 2:**
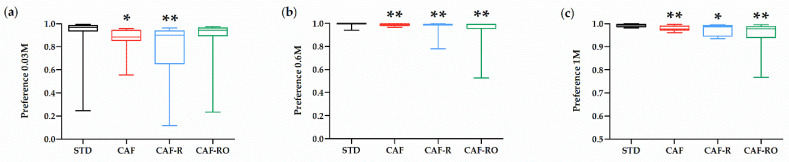
Effects of the dietary treatments on the sucrose preference at 0.03 M (**a**), 0.6 M (**b**) and 1 M (**c**) sucrose solutions in the two-bottle preference test. Sucrose preference ratio was calculated according to the formula: sucrose solution intake (g)/water + sucrose solution intake (g). Data are expressed as the mean ± SEM. * *p* < 0.05; ** *p* < 0.01 vs. STD group.

The preference ratios for sucrose solutions are represented in Figure 2. The STD group showed a greater preference for 0.03 M sucrose than the CAF and CAF-R groups, but not than the CAF-RO group (*p* < 0.05), which indicates a higher preference of CAF-RO group for low sucrose solutions compared with CAF and CAF-R groups. At higher concentrations (0.6 M and 1 M), the preference ratios of the STD group were also the highest and significantly different from all the other groups, whereas no differences were observed between CAF, CAF-R and CAF-RO groups (0.6 M *p* < 0.01; 1 M *p* < 0.05). There were no differences on preferences of the other sucrose concentrations among groups (0.01 M, 0.06 M, 0.1 M and 0.3 M).

#### 3.2.2. Total Fluid and Total Sucrose Intakes

To check if the water and sugar consumed through the diets might have influenced the sucrose and water intakes in the two-bottle preference test, we analyzed two more variables: (i) the total fluid intake (in mL), as the sum of intakes from the two bottles of the test plus the water intake from the CAF items consumed; and (ii) the total sucrose intake (in g), as the sum of sucrose consumption from sucrose bottle and the sugar intake from the CAF diet. The STD chow did not contain simple sugars.

**Figure 3:**
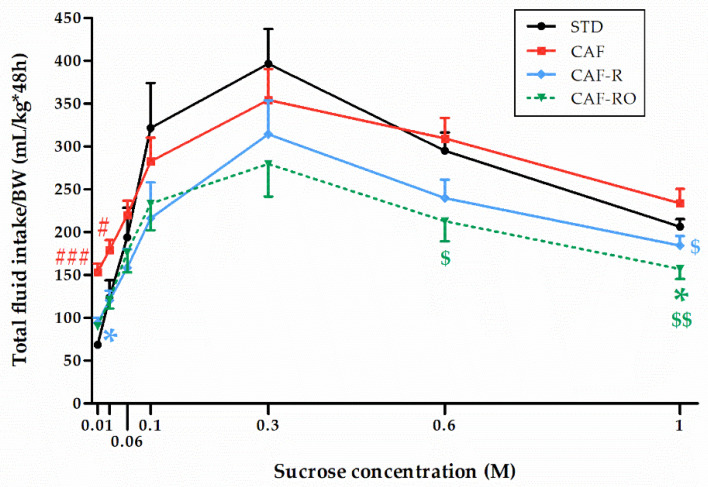
Effects of the dietary treatments on the total fluid intake during the two-bottle preference test. Total fluid intakes (mL) were relativized to the body weight (BW, kg). Data are expressed as the mean ± SEM. * *p* < 0.05 vs. STD group; $ *p* < 0.05, $$ *p* < 0.01 vs. CAF group; # *p* < 0.05, ### *p* < 0.001 vs. all groups.

The analysis of the total fluid intake (Figure 3) revealed a significant effect of concentration (*p* < 0.001), and that this effect was dependent on the dietary treatment (‘sucrose concentration × diet’, *p* < 0.05). The results revealed an overall inverted-U pattern similar to the one shown in Figure 1. Thus, STD and CAF animals consumed higher amounts of total fluid whereas the CAF-R and the CAF-RO groups showed lower consumptions and a similar pattern. Especially notable is the CAF-RO group, which significantly diminished the total fluid consumption at 0.6 M compared with the CAF group (*p* < 0.05), and at 1 M compared with both CAF (*p* < 0.01) and STD groups (*p* < 0.05). The CAF-R group also significantly diminished the total fluid intake at 1 M sucrose compared with the CAF group (*p* < 0.05).

**Figure 4:**
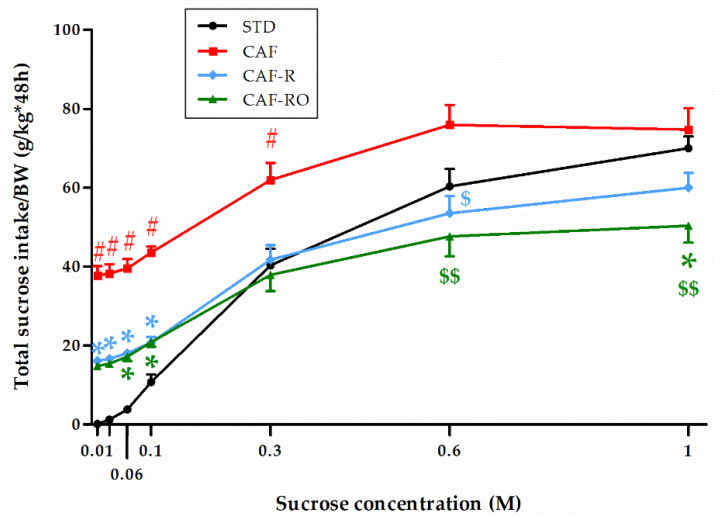
Effects of the dietary treatments on the total sucrose intake during the two-bottle preference test. Total sucrose intakes (g) were relativized to the body weight (BW, kg). Data are expressed as the mean ± SEM. * *p* < 0.05 vs. STD group; $ *p* < 0.05, $$ *p* < 0.01 vs. CAF group; # *p* < 0.05 vs. all groups.

Regarding the total sucrose intake (Figure 4), the results revealed a significant effect of concentration (*p* < 0.001), and that this effect was different depending on the dietary treatment (‘sucrose concentration × diet’, *p* < 0.001). The CAF-fed animals consumed the highest total amount of sugar over all sucrose concentrations. The STD group showed the lowest consumption values for the low sucrose concentrations although this increased to similar values to the CAF group at higher concentrations (0.6 M and 1 M). In contrast, both CAF-R and CAF-RO groups displayed intermediate values at low sucrose concentrations (0.01 M to 0.1 M) and the lowest intakes at higher concentrations (0.6 M and 1 M). Thus, at 0.6 M the CAF-R and CAF-RO groups showed lower values than the CAF group (*p* < 0.05), and at 1 M the CAF-RO group displayed lower intakes compared with both the CAF and STD groups (*p* < 0.05).

### 3.3. Taste Reactivity Test

**Figure 5:**
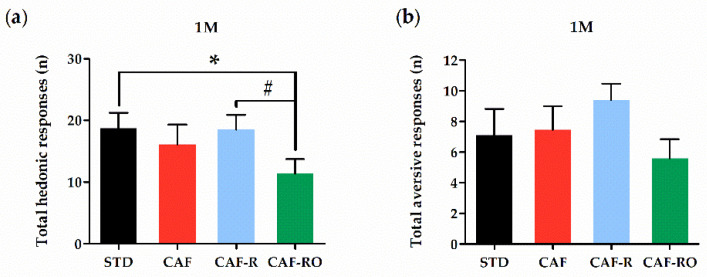
Total hedonic (**a**) and aversive (**b**) responses at the 1 M sucrose concentration in the taste reactivity test. The total number of hedonic responses was calculated as the sum of tongue protrusion, paw licking and mouthing responses. The total number of aversive was calculated as the sum of head shaking, forelimb flailing, snout cleaning and gagging responses. Data are expressed as the mean ± SEM. * *p* < 0.05 STD vs. CAF-RO group; # *p* < 0.05 CAF-R vs. CAF-RO group.

The analysis of the hedonic and aversive responses is shown in the Figure 5. The results revealed that the CAF-RO group exhibited a diminished number of total hedonic responses at the 1 M sucrose concentration compared with the STD and CAF-R groups (*p* < 0.05), whereas there were no differences on aversive responses among groups at this concentration. The analysis of hedonic and aversive responses to 0.1 M sucrose concentration showed no significant differences among groups (data not shown).

### 3.4. Correlations between Serum Leptin Levels and Sucrose Preference and Total Sucrose Intake

To study the relationship between serum leptin levels and sucrose preference we performed a Spearman’s rank correlation test (Table 2). The results showed that leptin levels were negatively correlated with sucrose preference for low (0.03 M, *p* < 0.01) and high sucrose concentrations (0.6 M and 1 M, *p* < 0.01).

**Figure 6:**
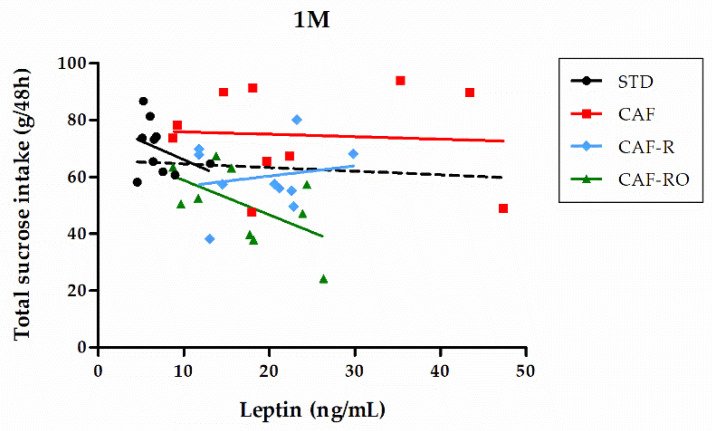
Correlation between serum leptin levels and total sucrose intake at the 1 M concentration. Solid lines represent the correlation trend line for each group (*n* = 10) and dashed line represents the correlation line for all animals in the study (*n* = 40).

The correlation between leptin and the total sucrose intake was analyzed for each group (Figure 6). We found that at the highest concentration (1 M), the STD and CAF-RO groups showed a negative correlation between both parameters (rs = −0.636, *p* < 0.05, and rs = −0.636, *p* < 0.05, respectively), whereas the CAF and CAF-R showed no correlation.

## 4. Discussion

In this study, the administration of the CAF diet for two months after weaning induced in the animals a greater increase in body weight and BMI, a higher food consumption, and metabolic alterations associated with obesity and MetS such as hypertriglyceridemia, insulin resistance and hyperleptinemia, compared with the STD diet. These results are in accordance with the obtained in our previous studies [16,27] and by other authors [17,18,19], confirming the validity of CAF diet as a preclinical model of DIO.

Once obesity was induced, we carried out two dietary interventions consisting of a calorie-restricted cafeteria diet and this same diet supplemented with an OLE polyphenolic extract. Three months after the dietary treatments, the group that continued CAF feeding showed similar alterations in biometric and metabolic parameters to those observed after the obesity induction period, whereas the CAF-R- and CAF-RO-fed groups reached similar body weights and BMIs to those of the STD chow-fed group. The food and energy intakes also decreased with these two dietary regimens, being intermediate between those of the CAF and STD groups. Thus, whereas the average energy intake of the CAF group was 216% of the STD group, those corresponding to the CAF-R and CAF-RO groups were 136% and 132%, respectively, of the STD group. As for the metabolic profile, only the CAF-RO diet restored serum levels of triacylglycerides and insulin resistance similar to the ones of the STD diet, which instead persisted at a higher level in the CAF-R group compared with the STD. Leptin levels remained higher in both cases compared with the STD group, although the differences between CAF-RO and STD were lesser than the differences between CAF-R and STD groups. Importantly, all the changes induced by the CAF-R and CAF-RO diets were achieved after 10 weeks of intervention. Further studies are needed to test the possibility of achieving better improvements with longer lasting dietary interventions.

One of our interests in the CAF-R diet was to simulate, at least in part, the human behavior of eating small portions of high-fat/high-sugar food or cafeteria food items from time to time while following dietary treatments to reduce body weight. In addition, a wide number of dietary obesity treatments are based on increasing the amount of healthy food eaten by the patient. In the same way, our CAF-R-fed rats increased their intake of the standard chow, here considered as the healthy food, and had limited access to small portions of the unhealthy cafeteria foods that were eaten *ad libitum* before the start of the dietary treatment. Thus, the CAF-R diet would be a useful animal model to investigate obesity reduction in obese subjects that commonly eat fast food, comfort food or cafeteria food. Additionally, the CAF-R diet provides the possibility to be supplemented with bioactive compounds such as OLE.

Other studies have reported a body weight reduction in rodents fed high-fat diets (HFD) supplemented with OLE. For example, Kuem et al. [23] administered the OLE with a HFD in the form of pellets during 10 weeks at a dose of 30 mg/kg*day and observed a reduction in body weight and an attenuated increase in body fat stores in mice. Our results showed that OLE did not enhance the effects of CAF-R diet alone on body weight gain and BMI, but it improved the metabolic profile. Interestingly, we found that the CAF-RO group showed diminished triglyceridemia and insulin resistance (HOMA-IR) compared with the CAF-R group and recovered normal levels showing no differences with respect to the STD group. These results are in line with other studies. Vezza et al. [32] have shown that oral gavage of different OLE doses (1, 10 and 25 mg/kg*day) for 5 weeks ameliorated body weight increase, decreased basal glycemia and insulin resistance, and improved endothelial dysfunction in mice fed a HFD. Notably, most of the beneficial effects of OLE were observed in the animals receiving the same dose administered in our study (25 mg/kg*day). Lepore et al. [22] also reported less weight gain and reduced HOMA-IR and serum lipids levels in mice fed a cafeteria diet after OLE supplementation at 20 mg/kg*day for 15 weeks. In addition, the nutraceutical supplementation of a HFD with OLE infused via syringe pump (758 mg/kg*day) for 10 weeks caused an important reduction of body weight increase, as well as normal serum glucose and leptin levels in mice [33]. The discrepancy between our results and these studies could be related to the fact that in the current study, OLE was added to a calorie-restricted cafeteria diet which improved obesity-associated biometric and metabolic parameters, in contrast with other studies where OLE was supplemented to HFD or cafeteria diets, which induce obesity and MetS in rodents. Thus, it cannot be discarded that higher doses and/or a longer-term intervention would be needed to further improve upon the CAF-R benefits per se. Additionally, other factors such as the administration route or the strain of rodents used may explain the different OLE effects reported among studies.

In this study, we also aimed to evaluate the effects of CAF diet and dietary interventions on sucrose intake and preference, as well as the possible changes in the sucrose-elicited hedonic reactions. The results of the two-bottle preference test indicated that CAF, CAF-R-, and CAF-RO-fed animals diminished sucrose intake compared with STD-fed animals over all sucrose concentrations tested. Consequently, the STD-fed animals consistently showed an increased sucrose preference, specifically for the concentrations 0.03 M, 0.6 M, and 1 M, compared to the cafeteria-fed groups. This would indicate a decreased sweet taste sensitivity induced by cafeteria feeding.

These results are consistent with previous studies evaluating taste functions associated with obesity. For example, rats fed high-energy diet for 4 weeks diminished sucrose (0.01 M–1 M) intake and preference compared with chow-fed rats [34]. Moreover, obesity-prone CAF-fed mice decreased sucrose preference relative to obesity-resistant CAF-fed mice [35]. Mice fed an obesogenic HFD for 12 weeks showed lower intake and preference for 1% sucrose solution compared with the chow-fed lean mice [36]. Rats and mice chronically subjected to an obesogenic high-fat diet become unable to detect properly low concentrations of sweet solutions during behavioral tests minimizing post-ingestive cues (e.g., neuro-endocrine regulations) [31]. Such a decrease of both peripheral detection and central perception to sweet stimuli might explain this relative loss of taste sensitivity, since sucrose-evoked calcium signaling has been shown to decrease in taste bud cells freshly isolated from DIO mice [37]. Concurrently, another study testing the intake and preference of the non-caloric sweetener saccharin reported that high-fat DIO rats diminished consumption and preference ratios for 0.01 M and 0.04 M saccharin solutions compared with normal diet rats [38]. Moreover, reductions of sweet solution intake and preference have been associated with diminished lingual gene expression level of the sweet taste receptor T1R3 in obesity-prone chow-fed rats compared with obesity-resistant chow-fed rats [34] and in high-fat-fed obese rats compared with chow-fed lean rats [38], which suggests that reduction of sweet solution intake and preference may be a consequence of altered sweet taste receptors.

In the aforementioned studies, rodents became obese because of the consumption of high-fat/high-sugar or cafeteria diets. Increased food intake and decreased taste or smell sensitivity in obesity appear to have parallel patterns in both animal [39] and human studies [40]. Obese patients have a greater predilection for sweet gustatory stimuli when compared with healthy controls [41] and a reduced hedonic response to sucrose or non-nutrient sweetener drinks in obese women compared with lean women [42]. However, whether this reduction in sweet perception is a metabolic consequence of obesity or the effect of drinking sucrose per se remains unclear [6].

As mentioned, the STD group displayed the highest sucrose preference. Ad libitum access to sucrose solutions could have induced an enhanced drive for consuming sweet solutions in STD fed rats compared to the animals from the other experimental groups since the former had their first experience with sweet taste during the two-bottle preference test that lasted 2 weeks. By contrast, the CAF and CAF-R fed rats already had daily sweet food experience from muffins, biscuits, and sugared jelly milk components of the cafeteria diet from a young age. Evidence from different animal models suggests that sensitivity and intensity of taste sensations changes with diet composition, thus involving a diet-dependent chemosensory plasticity that would result in changes to taste perceptions, their elicited responses and diet-related behavior (see May and Dus 2021 [7]). Evidence of dopamine (DA) neurotransmission changes associated with sugar consumption in sweet-naïve rats comes from Hajnal and Norgren 2001 [43] who, using microdialysis, reported that rats licking a 0.3 M sucrose solution showed a 305% increase in extracellular levels of DA and monoamine metabolites in the nucleus accumbens (NAcc) compared with water intake. Prolonged continuous consumption of a low concentration sucrose solution over 3 weeks also altered dopaminergic in addition to opioidergic systems of the NAcc [44]. Apparently, the changes in DA neurotransmission are related to the caloric value of sugar, since D2 antagonist raclopride increased the intake of the higher preferred sucrose solution in a two-bottle preference test but did not increase the intake of the higher preferred saccharin (non-caloric) solution [45]. Moreover, sweet taste is not the only driver of sucrose consumption, since sweet-blind trpm5-/- mice, lacking the cellular machinery required for sweet taste transduction, developed a robust preference for sucrose solutions based on caloric content and showed DA release in the ventral striatum in response to sucrose intake suggesting that calorie-rich nutrients can directly influence brain reward circuits that control food intake independently of palatability [46].

Other investigations pointed out that the changes in metabolic state also modulated the sensory pleasure of sweetness [47,48,49], and hedonic reactions of rats to sweeteners [50,51]. CAF-fed rats needed higher levels of brain stimulation to obtain the same hedonic responses as the STD chow-fed group; and chronic HFD elicits a down-regulation of DA and opioid receptors [52,53] in the mesolimbic area leading to a progressive devaluation of the reward value of oral stimuli, as found with abuse drugs [54]. Such a diet-acquired sensory deficiency might explain the tendency of DIO rodents to overeat highly rewarding foods [31], probably to gain the desired hedonic satisfaction [52].

An interesting recent study demonstrated that rats fed chronic HFD (42% fat) for 10 weeks post weaning showed a motivational impairment for sweet palatable foods, in terms of increased latency to start eating and diminished amount of ingested sweet palatable cereals or chocolate tablets [55]. In line with this, rats fed a HFD (60% fat) for 16 weeks showed diminished sucrose preference, increased anxiety and anhedonia [56], supporting that obese animals presenting anhedonia may lose their natural preference for sweet solutions [57].

To better understand the different pattern of responses obtained in the two-bottle preference test, we analyzed the total fluid and total sucrose intakes during the test, on the basis that the water and sugar consumed through the CAF diet may have played a role (no water and sugar are present in the STD chow). Our results revealed that during the preference test, the CAF group increased total fluid intake at the low 0.01 M and 0.03 M sucrose solutions whereas calorie-restricted cafeteria groups decreased total fluid intake at the high 0.6 M and 1 M sucrose solutions. Interestingly, this latter effect was even higher in the CAF-RO group which was the most significantly diminished and different from the standard chow group at 1 M. CAF-RO diet diminished the total fluid (water plus sucrose solution) intake compared to the CAF-fed animals across all concentrations. This result was also related to the one that emerged from the analysis of the total sucrose intake, referring to sucrose intake from the sucrose solution bottle plus sucrose from the diet. Thus, ad libitum cafeteria and calorie-restricted cafeteria feeding clearly increased total sucrose intake at low 0.01 M–0.1 M sucrose solutions, with the highest intake in the CAF-fed animals relative to all others, intermediate in the CAF-R- and CAF-RO-fed animals and the lowest in the STD group. This can be explained in accordance with the amount of sucrose diluted in the solutions and the dietary condition by itself, since CAF feeding included ad libitum sweet food; CAF-R and CAF-RO included a restricted amount, and no sucrose was present in the STD. We observed that the sucrose intake pattern varied among the dietary conditions and across the tested sucrose solutions. STD-fed animals progressively increased their intake from 0 to 70 g with the 1 M sucrose solution, which was very close to the 75 g consumed by the CAF-fed animals, whereas the CAF-R and CAF-RO groups progressively slowed down their consumption reaching an intake of 60 g and 50 g, respectively, with the 1 M solution, the CAF-RO group showing the smallest intake and lower than the STD group. Moreover, it is important to emphasize that even though the CAF-R and CAF-RO rats had the sucrose solution bottle available in their home cages to further increase sucrose intake, they did not, and their consumptions of 0.6 M and 1 M solutions were lower compared to the ones of the CAF and STD groups. All of this suggests that CAF-R and CAF-RO dietary treatments may have modified sweet taste and/or glucose regulation pathways. These effects could also be associated to the fact that these animals shifted from a CAF diet and its related metabolic alterations to the corresponding CAF-R or CAF-RO dietary treatment.

In accordance with these results, CAF-RO feeding reduced the number of hedonic responses to the 1 M sucrose solution in comparison to the STD and CAF-R groups, suggesting that this diet decreased its rewarding properties. This effect was not associated with an increase in the number of aversive responses. The lower preference and hedonic responses to the highest sucrose concentration due to the OLE supplementation could be related to the reported bitter taste of OLE and polyphenols in general [58]. However, in order to counteract this potential effect on taste, we diluted the OLE in 0.7% sugared fruit jelly, without compromising the quantity of sugars in the diet. We also took into account studies showing that functional polyphenols can be efficiently concentrated using gelatin, maintaining their functionality while absorbed [59], and that OLE can be added to the diet without modifying the hedonic taste of junk foods [60]. Moreover, polyphenols have been reported to be able to cross the blood–brain barrier and to localize in the whole central nervous system in a non-region-specific manner. OLE has also been shown to protect against oxidative stress in anesthetic-treated rats and to improve cognitive function [61]. Its antioxidant effect could account for a neuroprotective effect, which would promote the survival of DA neurons in the ventral tegmental area and therefore increase the availability of DA in the NAcc, improving sensitivity of the reward system [62].

Finally, our results showed that overall sucrose preference was negatively associated with serum leptin levels at 0.03 M, 0.6 M, and 1 M. Moreover, a negative association between total sucrose intake and serum leptin levels was specifically seen in the STD and CAF-RO groups but not in the CAF and CAF-R groups. There is evidence that leptin suppresses taste cell responses to sweet compounds [9] and that this action occurs by activation of the Ob-Rb leptin receptor in T1R3-positive taste cells [63]. Metabolic factors including high blood insulin or leptin resistance have been shown to enhance taste response to sweet stimuli orally administered [64,65]. All of this suggest that leptin may act as a modulator of sweet taste responses in mammals. Further studies are needed to elucidate the role of leptin in the changes in neural transduction of sweet taste in obesity.

## 5. Conclusions and Final Remarks

The administration of a 30% calorie-restricted diet (CAF-R) alone or supplemented with OLE attenuated body weight gain and BMI and diminished food and energy intake induced by CAF-feeding. As a new finding, OLE supplementation to a calorie-restricted diet improved some of the alterations in metabolic parameters, specifically triglyceridemia and insulin resistance. Additionally, it also partially restored sweet taste responses in obese animals such as the sucrose intake and its relationship with serum leptin levels, as well as the sucrose hedonic responses to the highest sucrose concentration. Although we must be cautious in extrapolating results from experimental models to humans, these results make us hypothesize that OLE may rescue the altered sweet taste alterations associated with the CAF diet-induced obesity, thus improving the function of the mesolimbic pathway of pleasure and reward and increasing sensitivity to sweet taste.

The main strengths of this study are the experimental design in terms of using a rodent model having considerable face validity with human obesity, the characterization of the effects of the CAF-R diet alone and supplemented with OLE, and the analysis of the intakes of the two-bottle sucrose vs. water preference test as well as the total sucrose intake and the taste reactivity responses. However, the use of only one OLE dose and the fact that the dietary intervention was not interrupted during the behavioral testing or that we did not include the sex female as an independent variable are limitations that would be of great interest to address in future studies.

## Figures and Tables

**Table 1:** Biometric, food intake and serum parameters.

CAF Diet-Induced Obesity Period			Dietary Treatments Period			
	STD	CAF	STD	CAF	CAF-R	CAF-RO
**Biometric parameters**	**Weeks 1–8**		**Weeks 9–19**			
Body weight gain (g)	255 ± 6	313 ± 5 +++	121 ± 5	158 ± 9 **	124 ± 5 $$	132 ± 6 $$
BMI (g/cm^2^) ^1^	0.64 ± 0.01	0.70 ± 0.01 ++	0.74 ± 0.01	0.83 ± 0.01 ***	0.78 ± 0.01 $	0.78 ± 0.02 $
**Daily food intake parameters**						
Food intake (g/kg)	101 ± 1	309 ± 2 +++	46.3 ± 0.6	140 ± 4 ***	77.2 ± 2.1 *** $$$	75.9 ± 1.3 *** $$$
Energy intake (kcal/kg)	292 ± 3	667 ± 5 +++	134 ± 2	289 ± 6 ***	183 ± 4 *** $$$	177 ± 4 *** $$$
Chow intake (kcal/kg)	292 ± 3	123 ± 3 +++	134 ± 2	44.1 ± 2.9 ***	58.8 ± 4.2 *** $	53.8 ± 4.5 ***
Simple sugars (kcal/kg)		196 ± 2		93.7 ± 3.6	36.8 ± 1.3 $$$	36.7 ± 1.1 $$$
**Serum parameters**	**Week 9**		**Week 19**			
Triacylglycerides (mmol/L)	0.93 ± 0.06	1.31 ± 0.09 +	1.07 ± 0.08	1.79 ± 0.22 **	1.58 ± 0.14 *	1.54 ± 0.13
Insulin (ng/mL)	0.51 ± 0.07	0.70 ± 0.05 +	0.61 ± 0.08	1.03 ± 0.17 *	0.82 ± 0.06	0.83 ± 0.10
HOMA-IR	3.20 ± 0.54	4.73 ± 0.37 +	3.68 ± 0.57	6.92 ± 1.31 *	5.65 ± 0.58 *	5.36 ± 0.76
Leptin (ng/mL)	4.23 ± 0.40	12.5 ± 1.0 +++	7.05 ± 0.78	23.7 ± 4.3 ***	19.1 ± 1.9 ***	17.0 ± 2.0 ***

Average daily food, energy and simple sugars intakes are relativized to the body weight (BW, kg). ^1^ BMI was calculated at week 9 and at week 19. STD, standard group; CAF, cafeteria diet group; CAF-R, calorie-restricted cafeteria diet group; CAF-RO, calorie-restricted cafeteria diet with OLE; HOMA-IR, homeostatic model assessment of insulin resistance. Data are expressed as the mean ± SEM. + *p* < 0.05, ++ *p* < 0.01, +++ *p* < 0.001 vs. STD group (obesity-induction period). * *p* < 0.05, ** *p* < 0.01, *** *p* < 0.001 vs. STD group; $ *p* < 0.05, $$ *p* < 0.01, $$$ *p* < 0.001 vs. CAF group (dietary treatments period).

**Table 2:** Correlation between leptin levels and sucrose preference (*n* = 40).

	0.01 M	0.03 M	0.06 M	0.1 M	0.3 M	0.6 M	1 M
rs value	−0.12	−0.38 *	−0.26	−0.13	−0.13	−0.44 **	−0.45 **
*p* value	0.45	0.02	0.10	0.44	0.43	0.01	0.01

* *p* < 0.05, ** *p* < 0.01 leptin vs. sucrose preference.
